# Acute liver failure in immunocompetent patients infected with hepatitis E

**DOI:** 10.1007/s00508-019-01547-2

**Published:** 2019-09-06

**Authors:** Christian Wenter, Wegene Borena, Georg Oberhuber, Ivo Graziadei

**Affiliations:** 1 Department of Internal Medicine, Academic Teaching Hospital Hall in Tirol, Milserstraße 10, 6060 Hall in Tirol, Austria; 2 Division of Virology, Medical University Innsbruck, Innsbruck, Austria; 3 INNPATH, Institute of Pathology, Tirol Kliniken Innsbruck, Innsbruck, Austria; 4 Medical University Innsbruck, Innsbruck, Austria

**Keywords:** Ribavirin, Zoonotic infection, Solid organ transplantation, Immunosuppression, Liver sausages

## Abstract

In almost all immunocompetent patients an acute hepatitis E virus (HEV) infection is clinically silent with spontaneous viral clearance. So far, only a very small number of severe acute HEV infections have been described. This article reports the case of a 78-year-old immunocompetent, diabetic patient, who presented with a symptomatic acute HEV genotype (GT) 3 infection and that progressed to acute liver failure. After starting with an antiviral therapy with ribavirin, the HEV viral load rapidly decreased with a significant improvement of the laboratory parameters as well as a clinical amelioration of the patient. The treatment was continued for 3 months and led to a complete resolution of this acute fulminant hepatitis E. Although the risk is almost negligible this article clearly demonstrates that an acute liver failure due to HEV should also be considered in immunocompetent patients, especially in older male individuals with diabetes mellitus.

## Introduction

While in the past hepatitis E virus (HEV) infection was mainly considered as a disease occurring almost exclusively in developing countries and as a travel-associated liver disease in western countries, an increasing number of autochthonous HEV infections have been observed in Europe in recent years [1]. Nowadays, HEV infection is the most common cause of acute viral hepatitis in many European countries. Based on recent seroprevalence data there are at least 2 million locally acquired HEV infections in Europe every year [2–4]. Whereas HEV genotypes (GT) 1 and 2 are obligate human pathogens spread by the fecal-oral route via contaminated water seen in areas with fragile sanitary infrastructure (e.g. Asia, Africa), in high-income countries hepatitis E is mostly a locally acquired zoonotic infection caused almost universally by HEV GT 3, with occasional cases caused by HEV GT 4. Beside HEV positive blood donations as an infection source, an increasing number of animals have been found to carry HEV. Pigs, wild boar and deer seem to be clinically relevant for human health caused by close contact or consumption of infected meat products, which have been undercooked or consumed raw [5].

In almost all immunocompetent patients, the course of the HEV infection is asymptomatic and self-limiting. In recent years only a very small number of cases with severe acute hepatitis progressing to liver failure have been reported; however, mainly in patients affected with the HEV genotype 1 and not with genotype 3 [6]. This article reports the case of an immunocompetent patient who presented with symptomatic acute hepatitis E (GT 3) and progressed to acute liver failure.

## Case presentation

In September 2018 a 78-year-old male patient was admitted to this hospital because of increasing itching and progressive fatigue over the previous days. The medical history included heart failure due to ischemic heart disease and an aorto-coronary bypass in 1990; furthermore, diabetes mellitus and arterial hypertension as well as chronic arterial fibrillation. The medication included metformin, lisinopril, bisoprolol and rivaroxaban, which was stopped immediately after admission. Diabetes mellitus was diagnosed in 2013 and was well-controlled. At admission the glycated hemoglobin (HbA1c) level was 5.4%. On examination, the patient’s vital signs were normal, the abdomen was soft without tenderness, but scleral jaundice was present. Blood results at admission showed significantly elevated aminotransferases and an increased total bilirubin. Liver synthetic functions were within normal limits. Other test results are shown in Table 1.

**Table 1:** Laboratory data of the patient on admission

Variable	Range	Admission	Start tx	5 days tx	Discharge	1 month tx	End of tx	3 months after tx D/C
Bilirubin direct (mg/dl)	0.01–0.1	2.15	12.97	19.37	19.83	5.38	0.83	–
Bilirubin total (mg/dl)	0.2–1.1	3.47	18.75	27.23	27.34	5.89	1.47	1.03
AST (U/l)	10–50	466	2080	413	219	31	16	19
ALT (U/l)	10–50	596	3044	1272	795	49	10	17
AP (U/l)	40–129	187	175	167	167	133	74	71
PT (%)	70–120	76	44	89	86	N. A.	40^a^	40^a^
INR	–	1.19	1.9	1.07	1.10	2.36^a^	1.89^a^	1.94^a^
Albumin (g/dl)	3.5–5.0	4.3	4.0	3.8	N. A.	3.6	4.0	4.5
Creatinine (mg/dl)	0.7–1.2	1.02	0.99	0.95	0.96	0.8	0.92	1.05
Hemoglobin (g/dL)	14.0–17.5	14.8	14.3	15.2	15.1	12.4	13.9	14.4
Platelets (10^9^/L)	150–350	205	359	387	374	273	234	225
LDH (U/l)	135–225	324	416	196	186	150	181	161
HEV-RNA (copies/ml)	–	–	7 × 10^12^	–	–	1.1 × 10^2^	N. D.	N. D.

^a^Oral anticoagulation therapy (rivaroxaban)

*AST* aspartate aminotransferase, *ALT* alanine aminotransferase, *AP* alkaline phosphatase, *LDH* lactate dehydrogenase, *INR* international ratio, *PT* prothrombin time, *tx* treatment, *D/C* discontinuation, *N.* *D.* not detectable, *N.* *A.* not available, *HEV-RNA* hepatitis E virus - ribonucleotide acid

Acute infections with hepatitis A, B and C as well as cytomegalovirus, Epstein-Barr virus, herpes simplex virus and varicella zoster virus could be excluded. Parameters suggesting an acute autoimmune hepatitis were also negative. The patient denied any consumption of special teas or herbs, in addition drug screening, including paracetamol level, were negative. Abdominal ultrasonography revealed a slight liver steatosis without signs of advanced fibrosis, a normal Doppler sonography of the portal and hepatic veins as well as the hepatic artery. Obstructive cholestasis as well as evidence of portal hypertension could be excluded. A multislice computed tomography (CT) scan did not add any further information. In addition, the non-alcoholic fatty liver disease (NAFLD) fibrosis score (−2.10) as well as the fibrosis(FIB)‑4 score (1.06) 2 months prior to admission excluded a pre-existing liver fibrosis.

Due to the still unclear etiology, a liver biopsy was performed indicating an acute hepatitis with portal and lobular inflammation and interface hepatitis but no signs of fibrosis (modified histological activity index, mHAI 9). According to these histological findings, the first differential diagnosis considered was a viral hepatitis but the pathologist could not exclude a drug-induced liver injury (DILI) due to the severity of the histological changes. The hepatitis E serological testing showed positive results for IgM and IgG antibodies (HEV ELISA IgM and IgG, MP Diagnostics, Marburg, Germany) with a high viral load of 7 × 10^12^ copies/ml confirming the diagnosis of a symptomatic acute HEV infection (RealStar HEV RT-PCR, Altona, Germany). Genotyping revealed the expected GT 3.

As acute HEV infections usually take a self-limiting course in immunocompetent patients with spontaneous clearance in almost all cases, the patient was placed under observation according to international guidelines [7]; however, during the following days liver function tests as well as liver synthesis parameters significantly deteriorated (see Table 1) and additionally, the patient developed hepatic encephalopathy (grade 2–3 according the West Haven classification) suggesting acute liver failure. Therefore, it was decided to commence ribavirin treatment at a daily dose of 1000 mg as recommended by the EASL guidelines [7].

The treatment with ribavirin was well tolerated and 5 days later a rapid decrease of the aminotransferases, a significant improvement of liver synthesis and of cognitive function were noticed. After 16 days the patient could be discharged in markedly improved clinical condition and was regularly followed-up in the outpatient clinic. At the end of the first month of treatment, the patient presented without any complaints, had normal aminotransferases and a significant improvement of the bilirubin levels, correlating with decreasing HEV-PCR values (see Table 1). After 3 months of antiviral therapy, HEV RNA was undetectable and therefore, ribavirin treatment was stopped. At the last follow-up visit in March 2019, the patient felt very comfortable, all laboratory parameters were within the normal range and the HEV PCR was still negative suggesting a complete virologic response and a complete recovery from the acute fulminant hepatitis E.

Regarding possible routes of infection, the patient denied any consumption of poorly cooked pork, wild boar or deer meat; however, he remembered consuming a mortadella sausage (local sausage speciality containing raw pork meat) some 6 weeks before admission. As there is evidence of high HEV prevalence in liver sausages and raw meat sausages [8], it was assumed that the consumption of a mortadella sausage was the route of infection in the patient.

## Discussion

Hepatitis E infection is an emerging global public health problem. Over the last years there has been a paradigm shift from an infection of developing countries (GT 1, 2) to an endemic locally acquired zoonotic infection (GT 3, 4) and HEV infections have become the major cause of acute viral hepatitis in many European countries [2]. In the majority of cases, acute HEV infections are asymptomatic with spontaneous clearance of the virus. Chronic courses with progression to liver cirrhosis and chronic liver failure were described only in immunosuppressed patients, in particular in solid organ transplant recipients. In contrast to these findings, the male patient in the present case developed a symptomatic acute HEV infection with GT 3 and progressed to acute liver failure. This case confirms a very small number of published data showing that older males are most likely to develop symptomatic hepatitis E affected with GT 3 or 4 [9]. The reason for this finding is still unclear. It is believed that underlying liver disease such as steatosis with or without fibrosis favors the development of clinically apparent hepatitis E, as shown in a study from England. In this study, most of the patients with HEV infection had concomitant diabetes mellitus or were heavy alcohol consumers, both risk factors for liver steatosis [10]. In accordance with these findings, the present patient suffered from diabetes mellitus for many years and liver steatosis was detected in ultrasound as well as in the CT scan. Acute HEV infections are usually self-limiting and do not require a specific antiviral treatment. Very few case reports, however, are available regarding ribavirin treatment for severe acute HEV infections. As this patient showed a rapid deterioration of liver function and, additionally, developed severe hepatic encephalopathy indicating progression to acute liver failure, it was decided to start treatment with ribavirin, although it cannot be proved retrospectively if the patient would have recovered without treatment. In accordance with the abovementioned cases [11], ribavirin treatment was associated with a rapid normalization of liver enzymes, an improvement of liver function and complete clearance of HEV-RNA in the patient. A European retrospective, multicenter study demonstrated that in patients with underlying hematological malignancies (42% with allogeneic hematopoietic stem cell transplantation) only early ribavirin treatment for acute HEV infections was associated with a better outcome [12]. It is assumed that ribavirin inhibits HEV replication by depleting guanosine triphosphate pools [13]. In solid organ transplant recipients, it has been shown that mycophenolic acid can be associated with spontaneous HEV clearance [6]. In individual patients with acute liver failure, who were retrospectively identified as HEV-related cases, corticosteroid treatment was associated with improved liver function parameters [14]; however, due to insufficient evidence, corticosteroid treatment cannot be supported for the treatment of HEV infection. Thus, ribavirin is the only currently established therapeutic option for HEV-induced acute or chronic hepatitis.

Frequently, it is difficult to identify the source of infection. Beside blood products, the consumption of undercooked or raw pork, wild boar or deer meat as well as liver is the main source for HEV infection in humans. Interestingly, the present patient consumed a mortadella sausage, which contains raw pork meat, approximately 6 weeks before admission. In a German study, HEV was found in about 10% of raw sausages and liver sausages in retail [15]. These data were confirmed by screening of ready to eat meat products for HEV in Switzerland demonstrating a HEV prevalence of 18.9% in liver sausages and 5.7% of raw meat sausages [8]. Therefore, the EASL clinical practice guidelines recommend avoiding the consumption of undercooked meat (e.g. pork, wild boar and venison) and shellfish in immunocompromized individuals and patients with chronic liver disease [7, 16]. For the general (immunocompetent) population, however, this recommendation is currently not justified and further data are needed, especially regarding older diabetic males with or without underlying liver disease [9, 10].

This article clearly demonstrates that all patients with acute hepatitis should be screened for hepatitis E infection. Additional studies are necessary to figure out which patients are especially prone to develop progressive liver disease and who might actually benefit from or show an urgent need for viral treatment.
